# Error Compensation for Area Digital Sun Sensor

**DOI:** 10.3390/s120911798

**Published:** 2012-08-29

**Authors:** Wen-Yang Li, Gao-Fei Zhang, Zheng You, Fei Xing

**Affiliations:** The State Key Laboratory of Precision Measurement Technology and Instruments, Tsinghua University, Beijing 100084, China; E-Mails: liwenyang09@mails.tsinghua.edu.cn (W.-Y.L.); yz-dpi@mail.tsinghua.edu.cn (Z.Y.); xingfei@mail.tsinghua.edu.cn (F.X.)

**Keywords:** Area APS Digital Sun Sensor, error factors, error compensation, calibration

## Abstract

Compared to the error factors of the Linear Array Digital Sun Sensor (DSS), those of the Area Array DSS are complicated and methods used for error compensation are not valid or simple enough. This paper presents the main error factors of the Area Array DSS and proposes an effective method to compensate them. The procedure of error compensation of Area Array DSS includes three steps. First, the geometric error of calibration is compensated; second, the coordinate map method is used to compensate the error caused by optical refraction; third, the high order polynomial-fitting method is applied to calculate the tangent of the sun angles; finally, the arc tangent method is used to calculate the sun angles. Experimental results of the product of the High Accuracy Sun Sensor indicate that the precision is better than 0.02° during the cone field of view (CFOV) of 10°, and the precision is better than 0.14° during the CFOV 10° to 64°. The proposed compensation method effectively compensates the major error factors and significantly improves the measure precision of the Area APS DSS.

## 1. Introduction

Sun Sensor, a device for satellite attitude control, is used to calculate the attitude angle between the sun and the satellite. The sun sensor, applied widely in various kinds of aerospace controllers, is one kind of common attitude control sensor [1]. According to their functions, sun sensors can be divided into 0–1 Sun Sensor, Analogue Sun Sensor (ASS), and Digital Sun Sensor (DSS) which is of high accuracy and large field of view (FOV). The image sensor in DSS is commonly the Charge Coupled Device (CCD) or Active Pixel Sensor (APS). In order to match the miniaturization of satellites, the satellite modules are required to be of minimal size, so sun sensors must have small size, light weight, and low power consumption. It is difficult to match these requirements with a CCD sun sensor, however. With the development of APS technology, the performance of APS image detector is catching up with and surpassing that of CCD image detectors, especially in cost, power consumption, system integration and the reading of signals. Therefore, more APS Sun Sensors are being installed in small satellites, microsatellites, and nano-satellites [2–5]. Due to its high accuracy, large field of view (FOV), small size, and low power consumption, the new Area Array APS DSS can measure the two axis sun angles.

The research on DSS focuses on system integration and the centroid algorithm of sun spots, without error compensation and calibration, which inevitably results in errors during the process of fixing. In order to achieve high measurement accuracy, it is necessary to research the error compensation of Area DSS. Using the method provided in thesis [6–13] to compensate the error of Area DSS, it is difficult to reach high accuracy when the two axis incident angles are both larger than 40°, and some methods are suitable for Linear Array DSS but are not suitable for Area Array DSS.

Based on the shortcomings of the above error compensation method, this study thoroughly analyzes the error factors and provides a means to compensate the error factors, especially (the) main error factors. In summary, our method compensates the geometry rotation error and optical refraction error respectively, followed by calculating the tangent values using a high order polynomial-fitting method to reduce the random error.

## 2. Measurement Theory

### 2.1. Measurement Model

The optical refraction caused by the surface protecting glass of the image detector results in the change of coordinate values that makes one-axis coordinate values in the same incident angle of the relative axis be different from the different incident angles of another axis. Therefore methods which are based on the theoretical measurement model, generate large errors between the measured and true values. Furthermore, the larger the incident angles are, the larger the errors are. In the FOV 64°, the largest error is 2°∼3°.

#### 2.1.1. The Model of Theoretical Measurement

**Figure 1.**
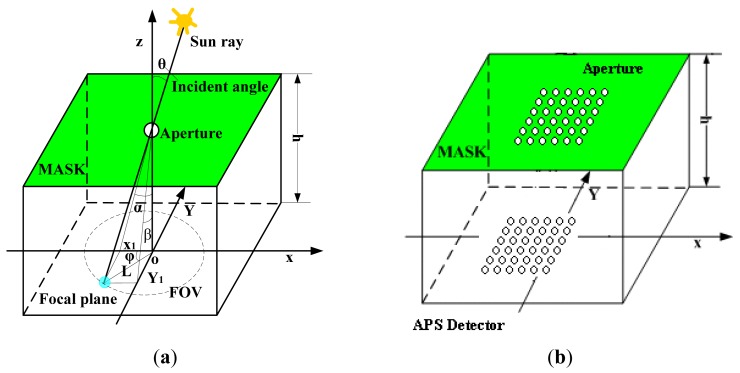
The principle of DSS. (**a**) Single aperture sun sensor; (**b**) Multiaperture sun sensor.

The principle of measuring Area DSS is shown in Figure 1. The principle of multi-aperture is the same as that of a single aperture sun sensor. Calculating the mean centroid coordinate value of apertures can improve the measurement accuracy. The incident sun rays create an image spot on the image detector. (*x_c_, y_c_*) are the coordinate values of the sun spot center, *l* is the distance from the sun spot center to the origin of the coordinate system, *h* is the distance between the surface of the image detector and the bottom of the optical mask glass, *θ* is the incident angle, and *α, β* are two-axis incident angles, pitch angle and lead angle, respectively.

According to the measurement model, it is not difficult to summarize the formula as follows:

(1) $$\alpha =\arctan(\frac{x_{c}}{h}),\beta =\arctan(\frac{y_{c}}{h}),\theta =\arctan(\frac{l}{h})$$

(2) $$l=\sqrt{x_{c}^{2}+y_{c}^{2}}$$

(3) $$\tan\theta =\sqrt{{\left(\tan\alpha \right)}^{2}+{\left(\tan\beta \right)}^{2}}$$

The feature of theoretical measurement model: when either *α* or *β* angle is fixed, the corresponding coordinate value of *x_c_* or *y_c_* becomes constant. The compensation method is based on a multiple-apertures digital sun sensor, and the mask has 36 apertures with the same distance between every two apertures. In the cone FOV 64°, the sun sensor calculates the mean centroid coordinate value of all the apertures.

#### 2.1.2. The Model of Optical Refraction Measurement

**Figure 2.**
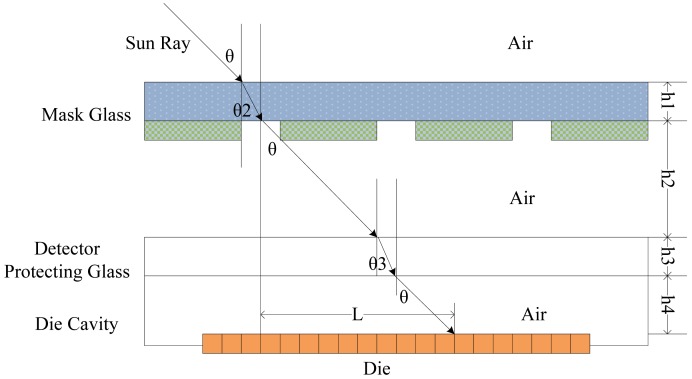
The optical refraction model of DSS.

In reality, sun rays have to pass through air, quartz glass, air, protecting glass of the image detector surface and air to the image detector surface, as shown in Figure 2, due to the presence of the image detector protecting glass and the optical mask glass. According to the model of theoretical measurement, it is obvious to summarize the formula as follows:

(4) $$l=(h_{2}+h_{4})\tan\theta +h_{3}\tan\theta_{3}$$

(5) $$n_{\text{glass}}=\frac{\sin\theta }{\sin\theta_{3}}$$

In formula (5), *n_glass_* is the refractive index of the protecting glass of the image detector.

According to the geometric projection rule, the two-axis coordinates are:

(6) $$x=l\times \cos\varphi ,y=l\times \sin\varphi ,\varphi =\arctan(\frac{\tan\beta }{\tan\alpha })$$

**Figure 3.**
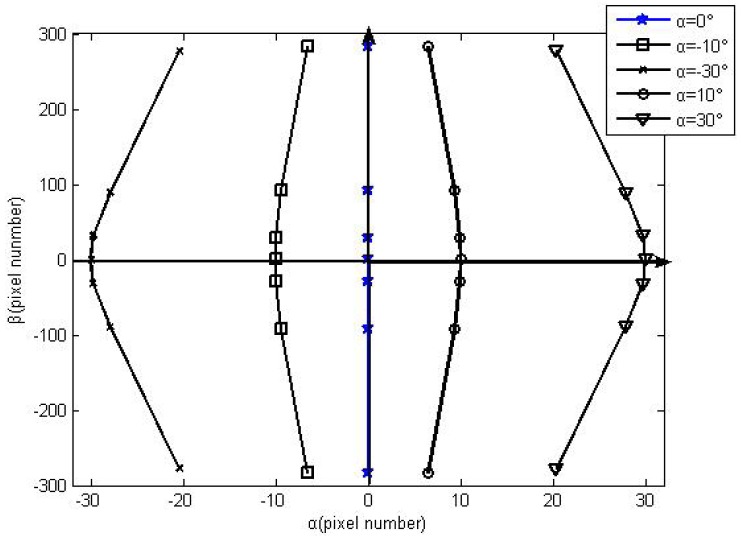
The trend of coordinate shift. In order to highlight the shift trend of the *x* coordinate, the *x* coordinate values translate towards the origin alone the *x* axis.

Formulas (4), (5) and (6) are combined to calculate the change of *x* coordinate values using Matlab, with the condition that the angle *α* is fixed, and the angle *β* is altered. The shift trend is shown in Figure 3.

From the shift trend, the shift of *x* coordinate values follows the shift of the angle *β* when the angle *α* is fixed. The shift trend of *x* coordinate values is contrary with that of the angle *β*. From the shift values, the largest error reaches 10 pixels in the FOV 64°. Therefore, the major error factor of DSS is caused by optical refraction of the protecting glass on the image detector surface.

### 2.2. The Calibration and Test Facility

Because of machining errors and fixing errors, the image detector surface deflects and rotates round the optical axis. DSS uses a calibration method to improve measurement accuracy. Our studies show it is ineffective to eliminate errors only through the fitting method.

**Figure 4.**
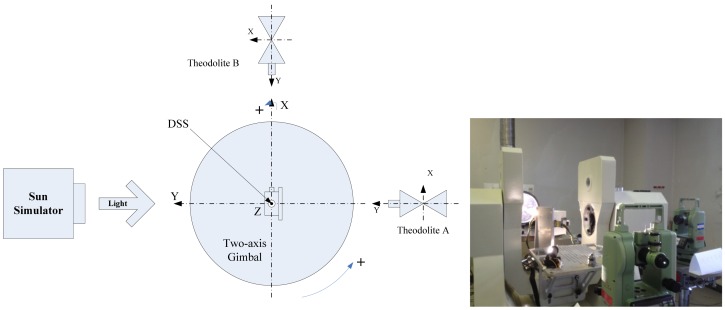
The calibration system schematic diagram and facilities of DSS.

DSS calibration facility (Figure 4) requires a sun simulator to supply a parallel light source whose brightness is equal to 1/10 solar constant, and a two-axis gimbal whose angular accuracy is 3 arc-seconds. Before the calibration, the platform of the gimbal was adjusted horizontally and the light from the sun simulator was also in the horizontal plane. The DSS was fixed on the gimbal. The two theodolites perpendicularly guarantee the DSS collinear with the sun simulator. The optical axis of the one theodolite is parallel to the light of the sun simulator. Then by rotating the gimbal according to a certain angle interval, arbitrary sun incident angles can be established, and the values of sun incident angles and the corresponding DSS output coordinates can be recorded. Each set of the angle values and corresponding coordinates are used to calculate the calibration factors.

## 3. Error Compensation Method

**Figure 5.**
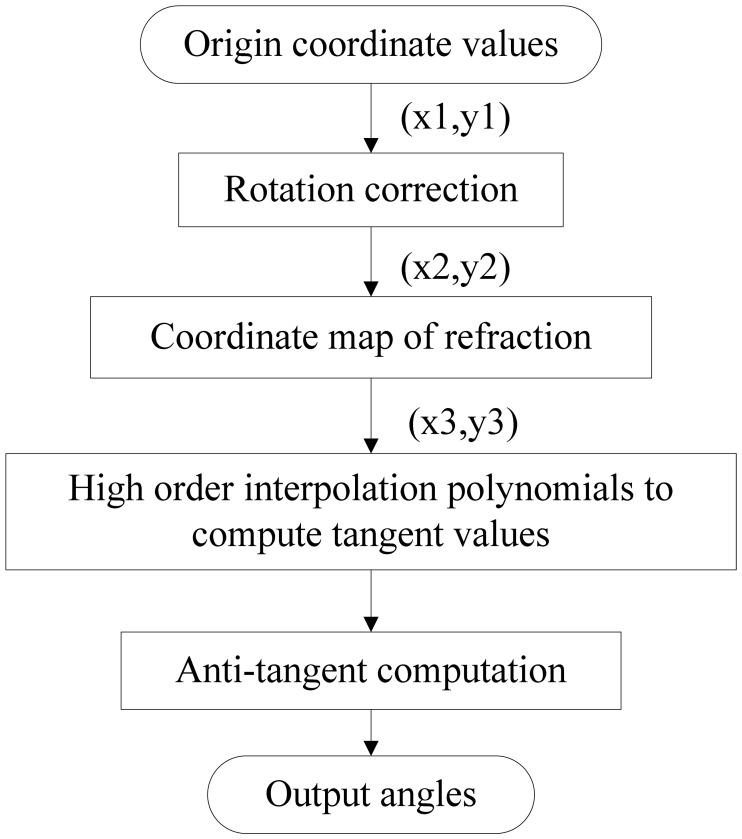
The flow chart of error compensation of DSS.

According to the above analysis, the main factors of error in DSS are optical refraction, and deflection and rotation of the image detector around the optical axis. Therefore, error compensated is used and its flow chart is presented in Figure 5.

### 3.1. The Correction for the Rotation Angle

#### 3.1.1. Calculation of the Rotation Angle

**Figure 6.**
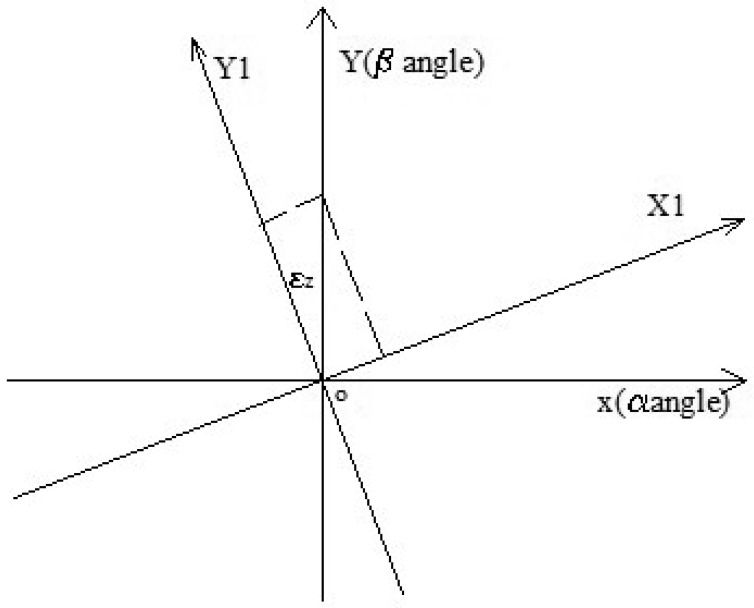
The sketch map of rotation of coordinate system.

Because of the rotation error and optical refraction, the coordinate value of the angle *α* is not 0 when the incident angle *α* is 0° and the incident angle *β* is not 0°. As shown in Figure 6, the axis *x* is relative to the angle *α*, and the axis *z* is relative to the angle *β*.

From the optical refraction rule as shown in Figure 3, it's known that refraction doesn't bring about shift of the coordinate value of the angle *α* when the incident sun rays move along the axis *y* (the angle *α* is 0°). For the rotation angle, the coordinate value of angle *α* is not 0 when the incident sun rays move along the axis *y* (the angle *α* is 0°). Besides, the coordinate of the angle *α* is symmetrical concerning the origin of coordinates. So the rotation angle can be calculated through the two-axis coordinate values when the angle *α* is 0°, and the angle *β* is from 0° to +64°.

According to the rule of coordinate system rotation, the formula to calculate the rotation angle is as follows:

(7) $$\varepsilon_{z}=\arctan(\frac{\left|x_{1}\right|}{\left|y_{1}\right|})$$

In order to the rotation angle more accurate, the arithmetic mean value of the rotation angle *ε_z_* is as the value for correlation.

#### 3.1.2. The Rotation Correction

The rotation angle *ε_z_* serves to calculate the coordinates *x*_2_, *y*_2_ without rotation error. According to the rule of coordinate system rotation, the formula is as follows:

(8) $$\left[\begin{array}{c} x_{2}\\ y_{2} \end{array}\right]=\left[\begin{array}{cc} \cos\varepsilon_{z} & \sin\varepsilon_{z}\\ -\sin\varepsilon_{z} & \cos\varepsilon_{z} \end{array}\right]\;\left[\begin{array}{c} x_{1}\\ y_{1} \end{array}\right]$$

In formula (8), *x*_1_, *y*_1_ are the coordinate values computed by the image processing chip of DSS, and *x*_2_, *y*_2_ are the coordinate values after the first rotation correlation.

Formula (9) is the equation used to compensate rotation error.

(9) $$\{\begin{array}{c} x_{2}=x_{1}\cos\varepsilon_{z}+y_{1}\sin\varepsilon_{z}\\ y_{2}=-x_{1}\sin\varepsilon_{z}+y_{1}\cos\varepsilon_{z} \end{array}$$

### 3.2. Coordinate Map of Refraction

#### 3.2.1. The method of Coordinate map

According to the analysis in Section 2.1.2, concerning the shift trend of coordinate values caused by refraction, the change of one-axis coordinate value depends on both the two-axis coordinate values. As a result, Dual-fit serves to map the coordinate values for correlation of optical refraction. The purpose of the map method is to make the coordinate values of the angle *α* under different values of the angle *β* equal to those of the angle *α* when the value of the angle *β* is 0. According to experimental results, the following Dual-fit map method can achieve high accuracy:

(10) $$\{\begin{array}{c} x_{3}=M_{1}x_{2}+M_{2}y_{2}+M_{3}x_{2}y_{2}+M_{4}\\ y_{3}=N_{1}x_{2}+N_{2}y_{2}+N_{3}x_{2}y_{2}+N_{4} \end{array}$$

In formula (10), *x*_2_, *y*_2_ are the coordinate values after the rotation correlation, and *x*_3_, *y*_3_ are the coordinate values after the coordinate map. In this paper, we collect 5° × 5° grid data points for calibration.

#### 3.2.2. Zoning for Map

From formula (10), the error influencing factor of the two coordinate axis values changes following the incident angles. It is difficult to achieve high accuracy if one group of map factors is taken into consideration in the whole CFOV. Therefore, map zoning is used, which reduces some random errors as well as produces less error fluctuations due to the identical coordinate ratio (*x/y*) in each strip-shaped area trend.

**Figure 7.**
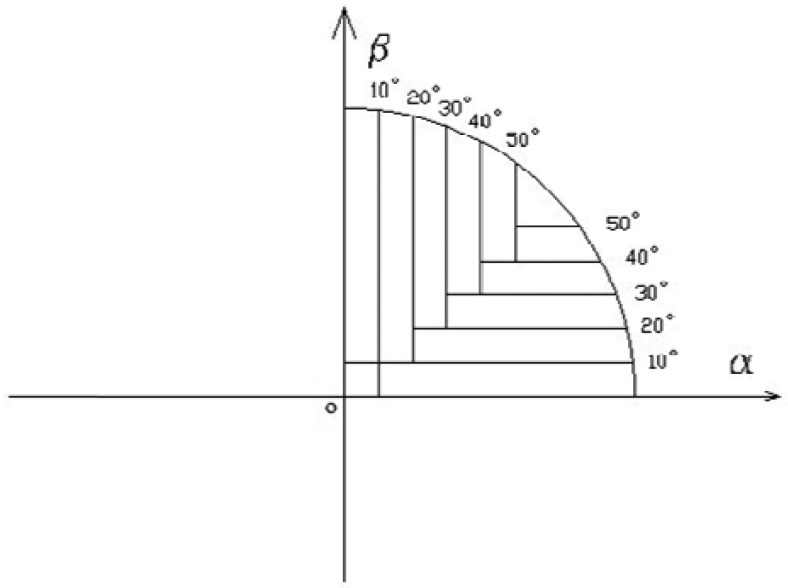
The sketch map of strip-shaped areas.

Zoning in the first quadrant is shown in Figure 7, dual-fit is made in every strip-shaped area of four quadrants. Every strip-shaped area has one group of map factors.

### 3.3. High Order Interpolation Polynomials

In order to reduce random errors such as deflection, the method of single axis high order interpolation polynomials serves to calculate the tangent values of one-axis angle. Then, the values of two-axis angles are calculated through an anti-tangent operation. The detailed method is as follows: the tangent values are calculated from the coordinate values either after the rotation correlation in the small CFOV or after the coordinate map correlation in the large CFOV.

The formula to calculate the tangent value is as follows:

(11) $$\alpha =\arctan(f(x))=\arctan(\sum_{i=0}^{n}a_{i}x^{i}),\beta =\arctan(f(y))=\arctan(\sum_{i=0}^{n}b_{i}y^{i})$$

In formula (11), *x* and *y* are the related coordinate values of the angle *α* and *β* after correlation, and *a_i_, b_i_* is the fit factors.

## 4. The Error Compensation Results

### 4.1. Experimental Test Case

**Figure 8.**
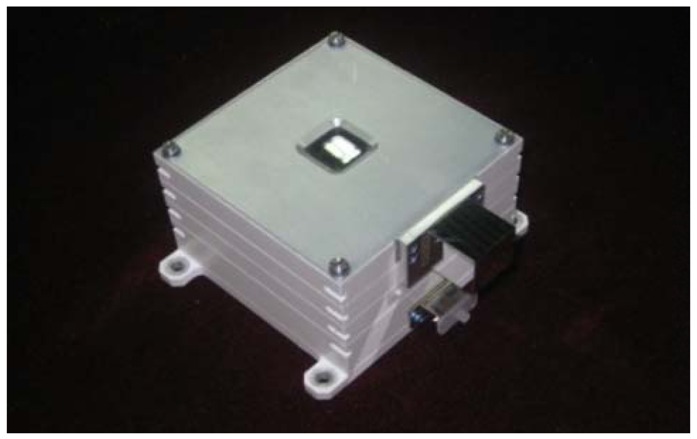
High Accuracy Sun Sensor with cubic prism.

The proposed method of error compensation was applied in a real High Accuracy Sun Sensor (HASS) product to be installed on board a satellite under development (Figure 8). The high accuracy sun sensor is an Area APS DSS, whose CFOV is 64°. The HASS image detector is a STAR1000 sensor with 1024 × 1024 pixels on a 15 μm pitch. The surface protecting glass of the STAR1000 sensor is BK7 glass, with a refractive index of 1.7.

**Figure 9.**
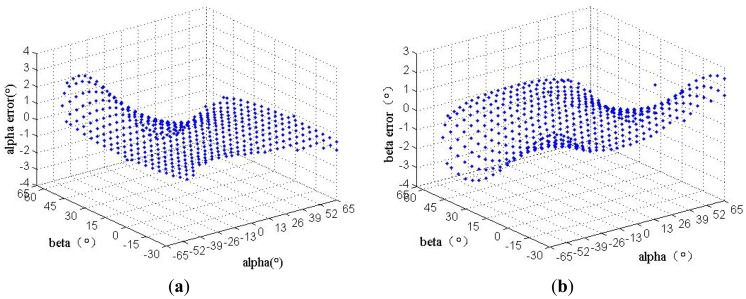
The error of HASS before compensation. (**a**) The error of *α* angle; (**b**) The error of *β* angle.

When HASS calculates the angle only through high order interpolation polynomials method, without using compensation, the measurement error is shown in Figure 9. The largest error surpassed 3°.

**Figure 10.**
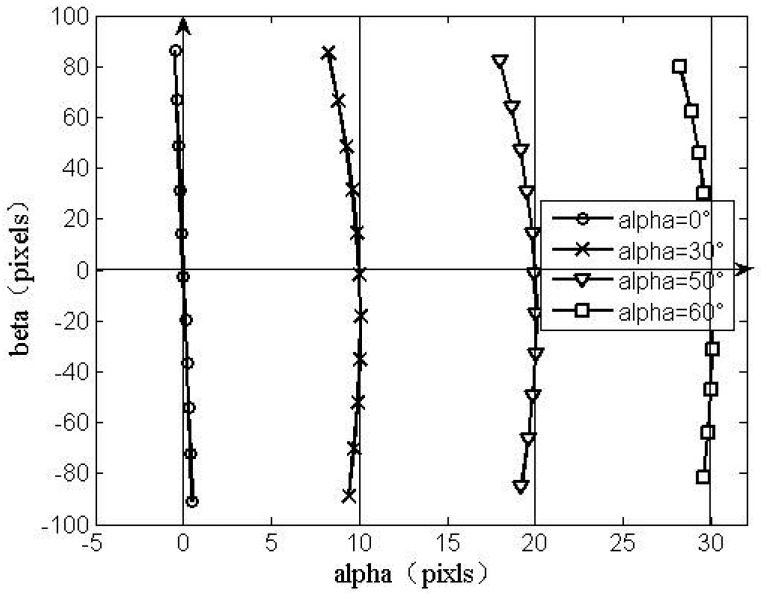
The coordinate shift trend of HASS before compensation.

Before using the proposed method to compensate HASS, the shift trend of coordinate values shown in the Figure 10 demonstrates the analysis in Sections 2.1.2 and 2.2.

### 4.2. The Compensation Index

Because the state of every sun sensor is not the same, the compensation index of every sun sensor is not same either.

#### 4.2.1. The Rotation Angle

The rotation angle is calculated through the coordinate values when angle *α* is 0° and angle *β* varies from 0 to 64° data in Table 1. The mean of the rotation angle $\bar{\varepsilon_{z}}=0.3226{}^{\circ}$.

#### 4.2.2. Index of Coordinate Map of Refraction

Based on a width of 10° of strip-shaped area, the map indexes of each strip-shaped area are listed in Tables 2 and 3.

#### 4.2.3. Index of Interpolation Polynomials

The calibration experiment collects the relative coordinate values of the incident angle. Then the calibration data including the coordinate value and angle value serves to calculate the index of high order interpolation polynomials in Matlab. Formula (12) computes the tangent value of incident angle as follows:

(12) $$\begin{array}{c} \tan\alpha =(0.0002)x^{5}+(-0.0026)x^{4}+(0.0138)x^{3}+(-0.0059)x^{2}+(0.4487)x+(-0.0002)\\ \tan\beta =(0.0002)y^{5}+(-0.0031)y^{4}+(0.0154)y^{3}+(-0.0077)y^{2}+(0.4492)y+(-0.0001) \end{array}$$

### 4.3. The Results of Error Compensation

**Figure 11.**
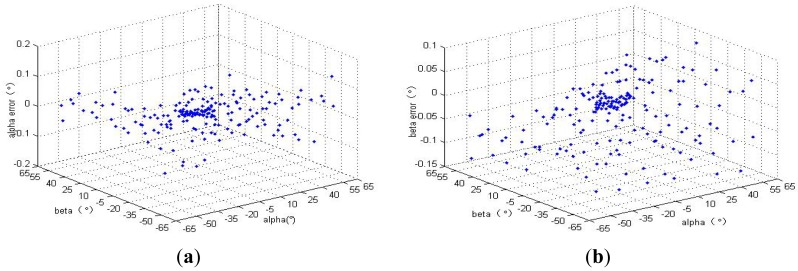
The error of HASS after compensation. (**a**) The error of *α* angle; (**b**) The error of *β* angle.

After compensation, the errors of the sun sensor in the cone FOV 64° (reference Figure 1) are listed in Table 4 and Figure 11. The measurement error in cone FOV 10° is smaller than 0.02° and that in FOV 10∼64° is smaller than 0.14°. And the test dataset includes 267 random points.

## 5. Conclusions

Previous works on Area Array DSS other than this paper do not undertake definite measurements to compensate for the two major error factors, which are the geometry error of fixing and the optical refraction caused by the surface protection glass of the image detector. Thus, it is difficult to attain high accuracy. According to the analysis mentioned above, the optical refraction is the most important error factor of DSS, and the method proposed in this paper compensates the geometry rotation error and optical refraction error, respectively.

HASS uses a high order interpolation polynomials method to compute the two-axis angles and produces a maximal error larger than 3°. By applying the proposed method of error compensation, the accuracy of HASS is greatly improved. The measurement error in FOV 10° is smaller than 0.02° and that in FOV 10°∼64° is smaller than 0.14°. The proposed method of error compensation is thus proven to be effective.

## Figures and Tables

**Table 1.** The values of rotation angle.

**α/°**	**β/°**	***x*_1_*_i_*/pixels**	***y*_1_*_i_*/pixels**	***ε_zi_*/°**
0	5	0.083	12.998	0.3634
0	10	0.155	25.850	0.3452
0	15	0.219	39.224	0.3211
0	20	0.302	53.020	0.3267
0	25	0.376	67.594	0.3183
0	30	0.458	83.249	0.3152
0	35	0.559	100.021	0.3201
0	40	0.659	118.644	0.3185
0	45	0.779	139.685	0.3194
0	50	0.898	164.061	0.3135
0	55	1.063	193.273	0.315
0	60	1.246	229.612	0.3109
0	64	1.429	266.858	0.3068

$\bar{\varepsilon_{z}}/{}^{\circ}$				0.3226

**Table 2.** Values of index M.

**strip Area/°**		**Index *M* × 10^3^**			

***α***	***β***	***M*_1_**	***M*_2_**	***M*_3_**	***M*_4_**
0–10	10–64	959.942	2.325	1.027	71.297
10–64	0–10	999.818	4.093	0.027	6.7755
10–20	10–64	972.889	7.332	0.645	−476.931
20–64	10–20	998.005	11.146	0.099	−164.034
20–30	20–64	951.478	6.945	0.671	458.043
30–64	20–30	988.327	8.890	0.228	415.243
30–40	30–64	953.791	17.295	0.520	465.957
40–64	30–40	981.513	22.595	0.216	545.475
40–50	40–64	957.596	34.327	0.332	698.877
50–64	40–50	968.727	38.571	0.234	518.038
50–60	50–64	967.158	50.032	0.190	317.087

**Table 3.** Values of index M.

**Angle Area/°**		**Index *N* × 10^3^**			

***α***	***β***	***N*_1_**	***N*_2_**	***N*_3_**	***N*_4_**
0–10	10–64	999.834	7.747	0.179	27.971
10–64	0–10	973.931	0.576	0.714	−16.651
10–20	10–64	997.435	5.043	0.135	39.810
20–64	10–20	970.039	0.067	0.653	216.528
20–30	20–64	990.156	9.915	0.217	275.415
30–64	20–30	964.378	4.041	0.597	355.476
30–40	30–64	983.609	21.922	0.221	350.355
40–64	30–40	966.815	15.921	0.450	107.906
40–50	40–64	979.153	38.063	0.180	124.986
50–64	40–50	968.361	34.565	0.265	133.359
50–60	50–64	971.270	41.640	0.200	458.288

**Table 4.** The max error in Field of view.

**Angle**	**The largest error of CFOV**	

	**<=10°**	**10°∼64°**
α	0.018°	0.138°
β	0.0193°	0.1208°
